# The prevalence, burden and risk factors associated with bronchial asthma in commonwealth of independent states countries (Ukraine, Kazakhstan and Azerbaijan): results of the CORE study

**DOI:** 10.1186/s12890-018-0676-7

**Published:** 2018-07-05

**Authors:** Damilya Nugmanova, Lyudmila Sokolova, Yuriy Feshchenko, Liudmila Iashyna, Olga Gyrina, Katerina Malynovska, Ilgar Mustafayev, Gulzar Aliyeva, Janina Makarova, Averyan Vasylyev, Luqman Tariq

**Affiliations:** 1 Semey State Medical University, Almaty, Kazakhstan; 2 National Institute of Phthisiology and Pulmonology F.G. Yanovsky of NAMS, Kiev, Ukraine; 3 National Medical University named after A.A. Bogomoltz, Kiev, Ukraine; 4 GlaxoSmithKline, Kiev, Ukraine; 5 Scientific Research Institute of Lung Diseases, Baku, Azerbaijan; 6 GlaxoSmithKline, Moscow, Russia; 7 GlaxoSmithKline, Dubai, UAE

**Keywords:** Bronchial asthma, Prevalence, Risk factors, Ukraine, Kazakhstan, Azerbaijan

## Abstract

**Background:**

In the Commonwealth of Independent States (CIS) countries epidemiology of Bronchial Asthma (BA) is poorly characterized. The objective of this analysis is to present the prevalence, burden and risk factors associated with BA in the CIS countries as part of the CORE study (Chronic Obstructive REspiratory diseases).

**Methods:**

A total of 2842 adults (≥18 years) were recruited (964 in Kiev, Ukraine, 945 in Almaty, Kazakhstan, and 933 in Baku, Azerbaijan) in 2013–2015 during household visits. A two-step cluster random sampling strategy was used. All respondents were interviewed about respiratory symptoms, smoking, medical history. Two definitions were used: (i) “doctor diagnosed asthma” when the respondent reported that he/she had ever been diagnosed with BA by a doctor, (ii) “wheezing symptoms” (when the respondent reported wheezing at the ATS Respiratory Symptoms Questionnaire during the study) using GINA (2012) recommendations. Chi-square tests were used to assess differences in proportions. Binary logistic regression was used to estimate odds ratios (OR) and 95% CI for association between risk factors and BA.

**Results:**

Prevalence of “doctor diagnosed asthma” was 12.5, 19.0 and 26.8 per 1000 persons, and prevalence of “wheezing symptoms” was 74.4, 254.8 and 123.4 per 1000 in Ukraine, Kazakhstan, and Azerbaijan, respectively. Statistically significant relationship with “wheezing symptoms” was shown for smoking (OR 1.99 (CI 1.22–3.27) in Ukraine, 2.08 (CI 1.54–2.81) in Kazakhstan, 8.01 (CI 5.24–12.24) in Azerbaijan); overweight/obesity (OR: 1.66 (CI 1.02–2.72); 1.94 (CI 1.44–2.62); 1.77 (CI 1.18–2.68), respectively) and dusty work (OR: 3.29 (CI 1.57–6.89); 1.68 (CI 1.18–2.39); 2.36 (CI 1.56–3.59), respectively), and for tuberculosis in Azerbaijan (OR: 10.11 (CI 3.44–29.69)). Co-morbidities like hypertension, cardiovascular diseases, abnormal blood lipids and a history of pneumonia occurred significantly (*p* < 0.05) more frequently in respondents with BA compared to those without BA across all participating countries.

**Conclusion:**

In CIS countries (Ukraine, Kazakhstan and Azerbaijan) the prevalence of doctor diagnosed asthma was significantly lower compared to prevalence of wheezing symptoms underlining that BA is likely to be underreported in these countries. The information provided in this paper will be helpful for healthcare policy makers in CIS countries to instruct BA management strategies and to allocate healthcare resources accordingly.

## Background

Bronchial asthma (BA) is prevalent throughout the world, with increasing prevalence particularly in developing countries, and associated with a high level of societal burden [1]. The rapid urbanisation in developing countries may be a potential cause for the increasing prevalence of BA related to increasing air pollution, decreasing exercise rates and stress resistance [2]. It is notably that over 80% of BA-related deaths occur in low and lower-middle income countries, where BA is often underdiagnosed and undertreated [3].

Despite of relatively low mortality rate compared to other chronic diseases, the main burden of asthma is disability, particularly among people aged less than 45 years; for people of older age groups and children premature death due to asthma also contributes to the burden of disease [1]. Based on the comprehensive analyses of the Global Burden of Disease Study undertaken in 2015 [4], BA is the most prevalent chronic respiratory disease, affecting an estimated 358 million people worldwide. Asthma affects people of all ages, but the prevalence of BA in middle-aged and older adults is much less known than in children [1].

Previously published studies evaluating the prevalence of BA have demonstrated that BA prevalence varies across different countries/populations [5–21]., Although the prevalence of BA has been estimated in CIS and post-soviet countries (e.g. Russia) as a part of global surveys or in separate studies, there is lack of large epidemiologic analyses of BA prevalence in the Commonwealth of Independent States (CIS) area. The largest epidemiologic survey evaluating BA prevalence in adults was the World Health Survey [5] performed in the early 2000s in 70 countries to evaluate the prevalence of self-reported doctor diagnosed asthma, clinical/treated asthma, and wheezing. The global prevalence rates were estimated at 4.3, 4.5, and 8.6% respectively. The prevalence varied by as much as 21-fold amongst the 70 countries, with the highest rate of asthma (all definitions) reported in Australia. In this study, the prevalence of doctor diagnosed asthma, clinical/treated asthma, and wheezing was estimated to 2.77, 2.90, 11.13%, respectively, in Ukraine; 1.43, 1.47, 3.36%, respectively, in Kazakhstan, and 2.50, 2.57, 4.98%, respectively, in Russia [5]. In Russia, an epidemiological study on adults was conducted in 2011–2012 using the GARD questionnaire. BA was defined based on symptoms (wheezing, or wheezing/whistling that resulted in breathlessness) or previously diagnosed (at some point in respondents’ life). Previous diagnosis of BA in this study was reported by 6.9% respondents, and 25.7% reported they ever experienced attacks of wheezing or whistling accompanied with the feeling of breathlessness [6].

Taking into account the lack of large epidemiologic analyses of BA prevalence in the CIS countries, the CORE study (Chronic Obstructive REspiratory diseases in CIS countries) has been conducted to address the gap in the epidemiological estimate of main chronic respiratory diseases and their risk factors in the selected cities of the CIS region. The aim of the CORE study was to evaluate country-specific prevalence of COPD (chronic obstructive pulmonary disease), BA and allergic rhinitis in order to obtain a clear “epidemiological picture” in selected CIS countries. In this paper, data obtained on the prevalence and burden of BA will be presented. Also, potential relationship between the presence of BA and its risk factors will be assessed.

## Methods

### Study area and population

Rationale and design of the CORE study (including key steps of the recruitment phase, inclusion and exclusion criteria, study population demographic characteristics, employment status, education and marital status of participants and questionnaires used in the study) have been described previously [22].

CORE study is a multinational, cross-sectional population-based epidemiological study carried out in major cities Kiev, Ukraine; Almaty, Kazakhstan; Baku, Azerbaijan, from the first half of 2013 till the end of 2015. The study enrolled subjects who were ≥ 18 years old, had ≥10 year of residence in selected city and provided a written informed consent for participation in the study.

Subjects who were not able to answer the study questionnaires (ATS Respiratory Symptoms Questionnaire, Alcohol Intake, Tobacco Smoking Questions) were excluded from the study. Subjects in whom spirometry could not be performed were also excluded (spirometry was required for COPD diagnosis in this study).

### Case definition

We used two definitions of asthma based on the study questionnaire and/or reporting wheezing as the ATS Respiratory Symptoms Questionnaire. Spirometry abnormalities alone were not classified as asthma diagnosis.

1. Doctor diagnosed asthma: When self-reported by the respondent (the respondent reported that he/she had ever been diagnosed with BA by a doctor while completing the study questionnaire). Respondents answered the following questions to identify “doctor diagnosed asthma”:

Has a doctor ever told you that you have bronchial asthma)?

If YES, please, indicate number of exacerbations during the last year.

2. Wheezing symptoms: When the respondent reported wheezing at the ATS respiratory symptoms questionnaire (answered positive to the question: “*Does your chest ever sound wheezy or whistling*?”).

### Data collection

The data were collected from participants during household visits. Study Executive Committee randomly selected streets for household visits in each city (country) applying stratified random cluster sampling procedure. Each city administrative district was divided into squares (on the map). 93 streets in each city were necessary to recruit 10 participants from a street (930 participants in total). The relevant number of streets in each district was determined proportionally to the district population. If the number of streets selected in a certain district was not a whole number, it was rounded down to the integer below, and additional number of streets (for the total number of 93) was obtained from the district(s), chosen randomly. A similar strategy was applied to map squares within districts, and the average number of streets per square to be sampled was determined. The streets within each map square for sampling were selected randomly by the Study Executive Committee.

Investigators (interviewers) performed household visits to collect the data. The interviewers visited households sequentially, starting with the 1st apartment of the 1st number of the house in the selected street, and continuing in ascending order. Data were collected from 10 subjects per street, and stratification by age was applied: 5 subjects aged 18–39 years old and 5 subjects aged ≥40 years old from the selected street were enrolled. When the potential participant was not available during the visit of interviewer, the interviewer performed up to three additional visits after 6 p.m. on working days and during all day on weekends.

At every household the interviewers assessed eligibility of all inhabitants. Participants who consented and were eligible for the study provided their socio-demographic information and medical history, underwent weight/height measurement and completed the study questionnaires, as described previously [22].

Socio-demographic data were collected to describe the characteristics of the overall study population, including gender, age and ethnicity distribution, body mass index (BMI), smoking status and alcohol intake. BA prevalence data were collected using the case definition described above and the impact of age, gender and severity was assessed. Additionally, the type and frequency of co-morbidities was investigated and association between BA and its related risk factors (i.e. smoking, BMI, alcohol intake, tuberculosis, dusty work, open fire cooking) was evaluated. Allergic rhinitis was identified as a part of the CORE study (when the respondent reported presence of watery runny nose (during the last 12 months) alone or in combination with any of the following: sneezing, nasal obstruction, nasal itching, or conjunctivitis at the Allergic Rhinitis Questionnaire), and will be subject of a separate publication.

### Statistical analysis

The prevalence of BA, as the number of BA individuals divided by total number of subjects included in the study, are expressed as a number per 1000 for each country. Prevalence was calculated in the subject population with valid data. 95% confidence intervals (CI) were calculated for each frequency using the Clopper-Pearson method [23]. Chi-square tests were used to assess differences in proportions. Binary logistic regression was used to estimate odds ratios (OR) and 95% CI for association between risk factors and BA. Statistical significance was assumed when *p* < 0.050. Statistical analysis was performed using IBM SPSS Statistics software (IBM Corp., USA) version 21.0 and R software version 3.1.2 (R Core Team, Austria).

## Results

### Study sample and demographics

A total of 2842 adult subjects were included in the CORE study (964 - in Ukraine, 945- in Kazakhstan and 933 – in Azerbaijan). Women were predominant across three countries: 58.2% in Ukraine; 63.2% in Kazakhstan and 58.3% in Azerbaijan. The mean age was slightly above 40 years in all participating countries. The majority of participants were Caucasian in Ukraine (99.7%) and Azerbaijan (100%), and almost two-third of participants in Kazakhstan were Asian (62.8%). The mean BMI was at the boundary of overweight in Ukraine, 25.0 (5.1) kg/m^2^, and in Kazakhstan, 25.7 (5.1) kg/m^2^, and it was 26.4 (5.3) kg/m^2^ in Azerbaijan. 33.7% participants in Ukraine, 40.2% in Kazakhstan, 26.0% in Azerbaijan were current or past smokers. See Table 1.

**Table 1:** Demographic characteristics of respondents

	Ukraine	Kazakhstan	Azerbaijan
Gender, n (%)			
Male	403 (41.8)	348 (36.8)	389 (41.7)
Female	561 (58.2)	597 (63.2)	544 (58.3)
Total	964 (100.0)	945 (100.0)	933 (100.0)
Ethnicity, n (%)			
Asian	3 (0.3)	593 (62.8)	0 (0.0
Black	0 (0.0)	1 (0.1)	0 (0.0
Caucasian	961 (99.7)	349 (36.9)	933 (100.0)
Other	0 (0.0	2 (0.2)	0 (0.0
Total	964 (100.0)	945 (100.0)	933 (100.0)
Age, years			
Mean (SD)	40.7 (15.1)	42.5 (15.3)	40.7 (14.8)
18–39 years old, n (%)	482 (50.1)	454 (48.0)	467 (50.1)
40–64 years old, n (%)	408 (42.4)	423 (44.8)	414 (44.4)
≥ 65 years old, n (%)	72 (7.5)	68 (7.2)	52 (5.6)
BMI, kg/m^2^			
Mean (SD)	25.0 (5.1)	25.7 (5.1)	26.4 (5.3)
Overweight/obesity (BMI ≥ 25 kg/m^2^), n (%)			
Overall population	437 (45.4)	449 (47.6)	511 (54.9)
Males	210 (52.1)	165 (47.6)	212 (54.5)
Females	227 (40.5)	284 (47.6)	299 (55.3)
Smoking: current/past smoker, n (%)			
Overall population	325 (33.7)	380 (40.2)	243 (26.0)
Males	191 (58.8)	227 (59.7)	232 (95.5)
Females	134 (41.2)	153 (40.3)	11 (4.5)
Alcohol intake, standard drinks^a^			
Mean (SD)	2.63 (4.15)	2.99 (7.54)	1.40 (2.82)
Alcohol intake category, n (%)			
Not at all	77 (8.0)	240 (26.3)	536 (57.4)
Moderate^b^	371 (38.6)	263 (28.9)	185 (19.8)
Heavy^b^	514 (53.4)	408 (44.8)	212 (22.7)
Dusty work^c^ (ever), n (%)			
Yes	63 (6.5)	178 (18.8)	199 (21.3)
No	901 (93.5)	767 (81.2)	734 (78.7)

^a^ One drink was defined as 12 fluid ounces of regular beer (5% alcohol), 5 fluid ounces of wine (12% alcohol), or 1.5 fluid ounces of 80 proof (40% alcohol) distilled spirits. One drink contains 0.6 fluid ounces of alcohol

^b^ Moderate alcohol consumption was defined as the consumption of up to 1 drink per day for women and up to 2 drinks per day for men. Heavy (or high-risk) drinking was defined as the consumption of more than 3 drinks on any day or more than 7 per week for women and more than 4 drinks on any day or more than 14 per week for men

^c^ Respondents answered (“YES” or “NO”) the question: “Have you ever worked for a year or more in any dusty job?”

### Prevalence of BA

Subjects who fulfilled the definitions used in this study for BA are shown in Table 2.

**Table 2:** Number of respondents with doctor diagnosed asthma and wheezing symptoms identified during the study

	Ukraine (*N* = 939)	Kazakhstan (*N* = 945)	Azerbaijan (*N* = 933)
Doctor diagnosed asthma (ever diagnosed by a doctor in the past and reported by the respondent while completing the study questionnaires)			
Overall population, n (%)	12	18	25
Males / Females, n (%)	7 / 5	7 / 11	12 / 13
Age category 18–39 / 40–64 / ≥65, n (%)	6 / 2 / 4	6 / 9 / 3	11 / 4 / 0
Caucasians / Asians, n (%)	12 / 0	6 / 12	25 / 0
Wheezing symptoms (answered positive to the question at the ATS Respiratory Symptoms Questionnaire: “*Does your chest ever sound wheezy or whistling*?”)			
Overall population, n (%)	70	237	115
Males / Females, n (%)	30 / 40	100 / 137	88 / 27
Age category 18–39 / 40–64 / ≥65, n (%)	24 / 30 / 16	91 / 125 / 21	39 / 71 / 5
Caucasians / Asians, n (%)	69 / 1	101 / 136	115 / 0
BA exacerbations during the last year (self-reported by the respondent who had “doctor diagnosed asthma”)			
Overall population, n (%)	9	9	19

**Fig. 1:**
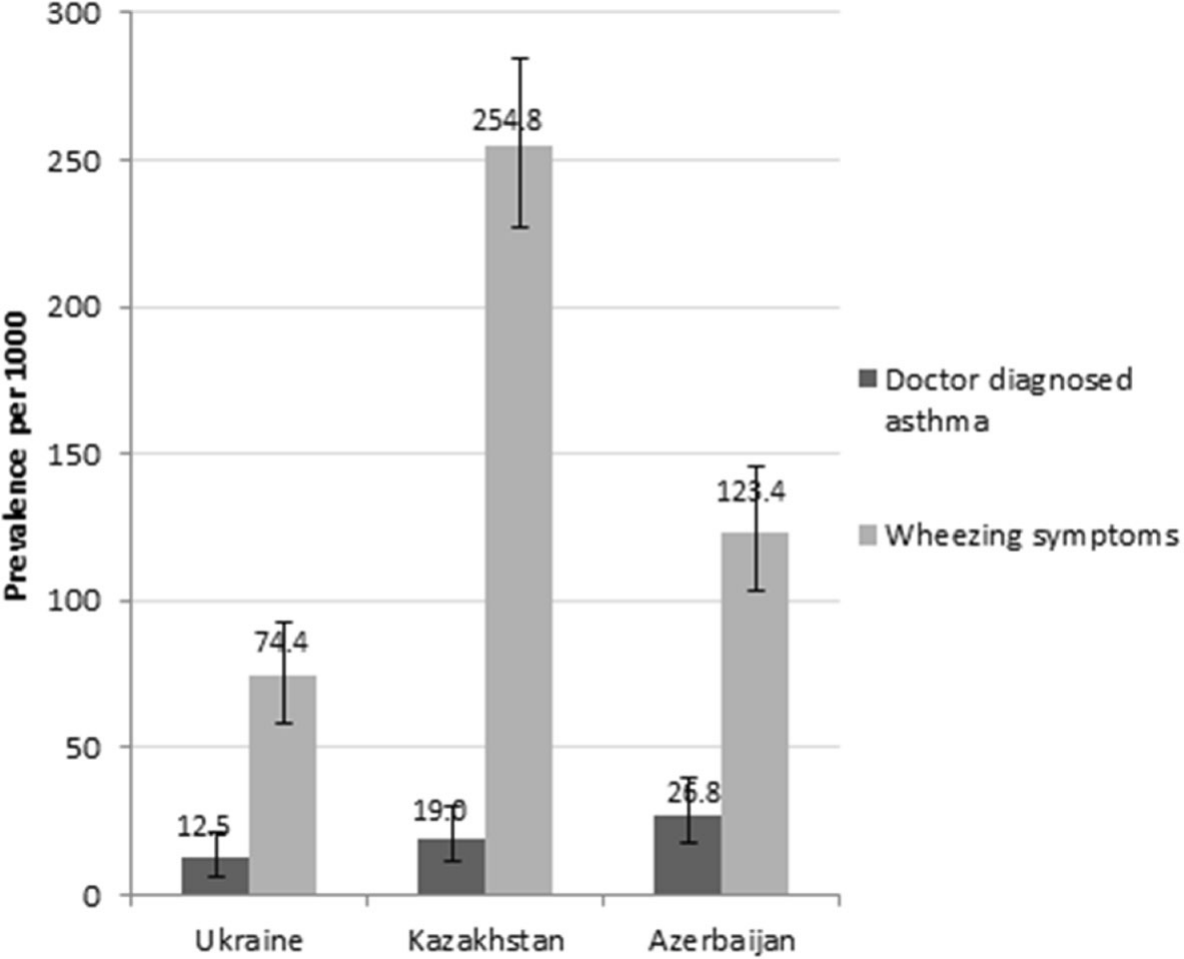
Prevalence of doctor diagnosed asthma and wheezing symptoms (prevalence per 1000 and 95% confidence intervals). The prevalence was calculated per 1000 persons and expressed with 95% confidence intervals, for two definitions: *doctor diagnosed asthma*: when self-reported by the respondent (the respondent reported that he/she had ever been diagnosed with BA by a doctor while completing the study questionnaire), and *wheezing symptoms*: when the respondent reported wheezing at the ATS Respiratory Symptoms Questionnaire (answered positive to the question: “*Does your chest ever sound wheezy or whistling*?”)

The estimated prevalence of wheezing symptoms among the adult population was 74.4 (95% CI 58.4–93.1) per 1000 in Ukraine, 254.8 (95% CI 227.1–284.1) per 1000 in Kazakhstan and 123.4 (95% CI 103.0–146.2) per 1000 in Azerbaijan. The prevalence of doctor diagnosed asthma was lower than the prevalence of wheezing symptoms: 12.5 (95% CI 6.5–21.7), 19.0 (95% CI 11.3–29.9) and 26.8 (95% CI 17.4–39.4) per 1000, in Ukraine, Kazakhstan and Azerbaijan, correspondingly. See Fig. 1.

We estimated the BA prevalence in the subgroups by age, sex or ethnicity, when possible, as seen in Table 2.

**Fig. 2:**
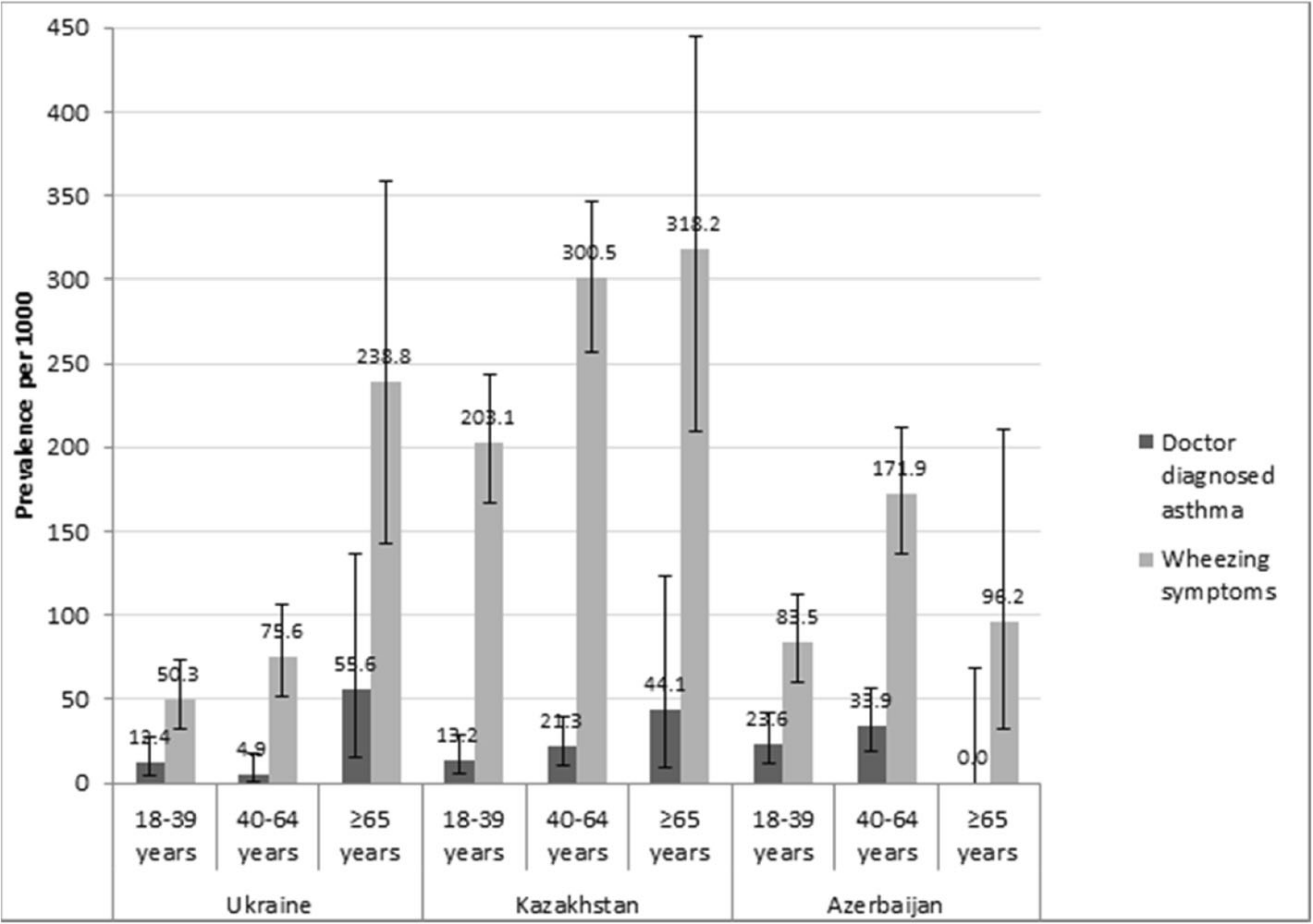
Prevalence of doctor diagnosed asthma and wheezing symptoms stratified by age (prevalence per 1000 and 95% confidence intervals). The prevalence of BA was calculated per 1000 persons and expressed with 95% confidence intervals in three age groups: 18–39, 40–64, and ≥ 65 years old, for *doctor diagnosed asthma*: when self-reported by the respondent (the respondent reported that he/she had ever been diagnosed with BA by a doctor while completing the study questionnaire), and *wheezing symptoms*: when the respondent reported wheezing at the ATS Respiratory Symptoms Questionnaire (answered positive to the question: “*Does your chest ever sound wheezy or whistling*?”)

The following prevalence values of doctor diagnosed asthma were observed in the age group ≥65 years old in Ukraine and Kazakhstan: 55.6 (95% CI 15.3–136.2) per 1000 and 44.1 (95% CI 9.2–123.6), respectively; in age group 40–64 years old in Azerbaijan: 33.9 (95% CI 18.7–56.2). The following prevalence values of wheezing symptoms were observed in the population aged ≥65 years in Ukraine and Kazakhstan: 238.8 (95% CI 143.1–358.6) per 1000 and 318.2 (95% CI 208.9–444.4) per 1000, respectively, and in age group 40–64 years old in Azerbaijan: 171.9 (95% CI 136.8–211.8) per 1000. See Fig. 2.

**Fig. 3:**
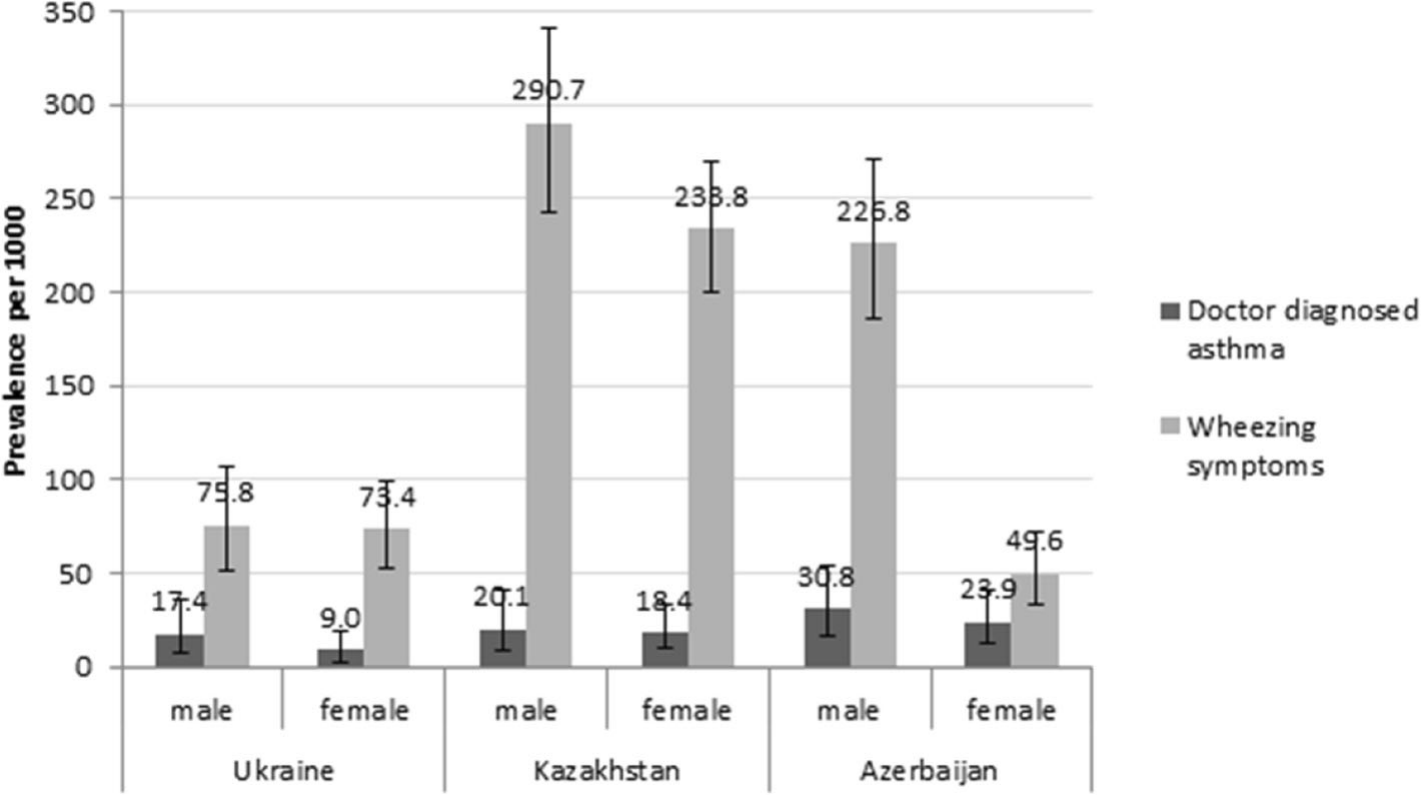
Prevalence of doctor diagnosed asthma and wheezing symptoms stratified by sex (prevalence per 1000 and 95% confidence intervals). The prevalence of BA was calculated per 1000 persons and expressed with 95% confidence intervals, among males and females, for *doctor diagnosed asthma*: when self-reported by the respondent (the respondent reported that he/she had ever been diagnosed with BA by a doctor while completing the study questionnaire), and *wheezing symptoms*: when the respondent reported wheezing at the ATS Respiratory Symptoms Questionnaire (answered positive to the question: “*Does your chest ever sound wheezy or whistling*?”)

The prevalence of doctor diagnosed asthma was 17.4 (95% CI 7.0–35.6) per 1000 in males vs 9.0 (95% CI 2.9–20.8) per 1000 in females in Ukraine; 20.1 (95% CI 8.1–41.0) per 1000 in males vs 18.4 (95% CI 9.2–32.7) per 1000 in females in Kazakhstan; 30.8 (95% CI 16.0–53.3) per 1000 in males vs 23.9 (95% CI 12.8–40.6) per 1000 in females in Azerbaijan. The prevalence of wheezing symptoms was 75.8 (95% CI 51.7–106.4) per 1000 in males vs 73.4 (95% CI 52.9–98.6) per 1000 in females in Ukraine; 290.7 (95% CI 243.2–341.8) per 1000 in males vs 233.8 (95% CI 200.1–270.2) per 1000 in females in Kazakhstan; 226.8 (95% CI 186.1–271.8) per 1000 in males vs 49.6 (95% CI 33.0–71.4) per 1000 in females in Azerbaijan (Fig. 3).

The ethnicity-specific prevalence of BA was estimated only in Kazakhstan, because the majority of Ukrainians and Azerbaijanians were Caucasians. The prevalence of doctor diagnosed asthma was 20.2 (95% CI 10.5–35.1) per 1000 in Asians and 17.2 (95% CI 6.3–37.1) per 1000 in Caucasians. At the same time, prevalence of wheezing symptoms was 295.3 (95% CI 247.5–346.8) per 1000 in Caucasians and 232.1 (95% CI 198.5–268.4) per 1000 in Asians.

### BA description at the ATS respiratory symptoms questionnaire

Analysis of the ATS Respiratory Symptoms Questionnaire (section “Wheezing”) showed that among respondents who answered positive to the question: “*Does your chest ever sound wheezy or whistling*?” 49/70 (Ukraine) 117/237 (Kazakhstan), and 91/115 (Azerbaijan) had wheezy or whistling during the last 12 months. 52, 147, 109 respondents in Ukraine, Kazakhstan, Azerbaijan, respectively, reported the duration of their symptoms. Interestingly, among them, 22/52 respondents in Ukraine, 64/147 in Kazakhstan and 56/109 in Azerbaijan reported symptoms duration > 5 years; others reported shorter duration. For reference, as displayed in Table 2, only 12, 18 and 25 respondents, respectively, reported about doctor diagnosed BA, that additionally point at the underdiagnosis of BA in the studied countries.

### Risk factors associated with BA

**Fig. 4:**
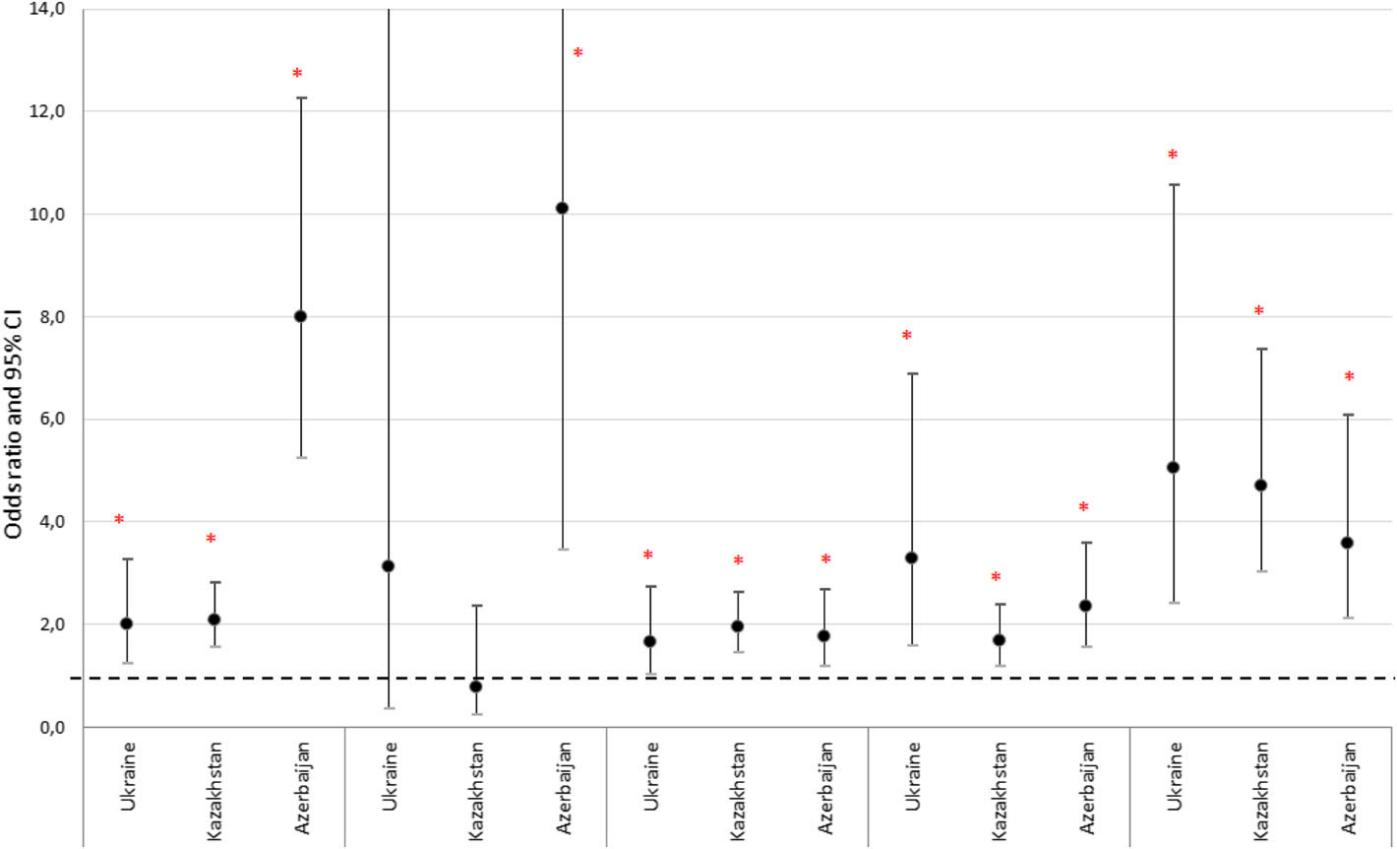
Association between risk factors and wheezing symptoms (odds ratios [OR] and 95% confidence intervals for OR). Odds ratios [OR] and 95% confidence intervals for OR are presented for each potential risk factor. Asterisk (*) means statistically significant association between risk factor and wheezing symptoms (*p* < 0.05)

Relationship between the presence of BA (wheezing symptoms) and smoking status (current/past smoker), alcohol intake (heavy alcohol drinking, i.e. consumption of more than 3 drinks on any day or more than 7 per week for women and more than 4 drinks on any day or more than 14 per week for men), BMI (BMI ≥ 25 kg/m2), tuberculosis (ever been diagnosed), dusty work, open fire cooking and allergic rhinitis was investigated and statistical significance was found between wheezing symptoms and smoking in Ukraine (OR 1.99 (CI 1.22–3.27) *p* = 0.005), Kazakhstan (OR 2.08 (CI 1.54–2.81) p ˂0.001), Azerbaijan (OR 8.01 (CI 5.24–12.24) p ˂0.001); between BA and overweight/obesity in Ukraine (OR 1.66 (CI 1.02–2.72) *p* = 0.041), Kazakhstan (OR 1.94 (CI 1.44–2.62) *p* < 0.001), Azerbaijan (OR 1.77 (CI 1.18–2.68) *p* = 0.006); between BA and dusty work in Ukraine (OR 3.29 (CI 1.57–6.89) *p* = 0.001), Kazakhstan (OR 1.68 (CI 1.18–2.39) *p* = 0.004), Azerbaijan (OR 2.36 (CI 1.56–3.59) *p* < 0.001); between BA and tuberculosis in Azerbaijan (OR 10.11 (CI 3.44–29.69) *p* < 0.001); and between wheezing symptoms and allergic rhinitis in Ukraine (OR 5.05 (CI 2.42–10.55) *p* < 0.001), Kazakhstan (OR 4.71 (CI 3.02–7.36) p < 0.001), Azerbaijan (OR 3.58 (CI 2.12–6.06) *p* < 0.001). See Fig. 4.

### Co-morbidities

While completing the study questionnaire the respondents were asked to report the presence of other chronic medical conditions except for respiratory diseases. Co-morbidities were reported by 44.2% respondents in Ukraine, 23.5% respondents in Kazakhstan and 54.6% respondents in Azerbaijan. The respondents with BA (respondents who reported wheezing symptoms at the ATS Respiratory Symptoms Questionnaire, according to study definition) were compared to the respondents without BA by the rate of co-morbidities. The rate of co-morbidities in the respondents with BA was higher (*p* < 0.05) than in those without BA (63% vs 42% in Ukraine, 51% vs 21% in Kazakhstan, and 77% vs 51% in Azerbaijan, respectively), especially for hypertension, cardiovascular diseases, abnormal blood lipids and a history of pneumonia. Allergic rhinitis (which was identified as a part of this study as a presence of watery runny nose (during the last 12 months) alone or in combination with any of the following: sneezing, nasal obstruction, nasal itching, or conjunctivitis) was also observed more frequently (*p* < 0.001) in the respondents with BA compared to those without BA in all countries. See Table 3.

**Table 3:** Co-morbidities in the respondents with and without BA

	Ukraine			Kazakhstan			Azerbaijan		
	Respondents^a^			Respondents^a^			Respondents^a^		
	without BA	with BA	*p*-value	without BA	with BA	*p*-value	without BA	with BA	*p*-value
Valid N	871	70		693	237		817	115	
Any co-morbidity, %	42.0	62.9	0.001	20.8	50.6	< 0.001	51.4	77.4	< 0.001
Hypertension, %	13.1	37.1	< 0.001	21.6	40.1	< 0.001	16.2	27.0	0.006
Diabetes, %	3.2	5.7	0.291	3.5	4.2	0.689	4.8	9.6	0.044
Cardiovascular disease, %	6.0	14.3	0.013	7.0	13.9	0.001	3.5	8.7	0.015
Abnormal blood lipids, %	4.3	10.0	0.039	10.0	17.3	0.004	1.0	3.5	0.050
Depression, %	0.5	1.4	0.322	0.4	0.8	0.607	1.7	3.5	0.263
Anxiety, %	0.2	0.0	1.000	0.6	0.4	1.000	2.6	3.5	0.758
Osteoporosis, %	0.6	1.4	0.374	1.5	2.5	0.384	0.5	0.9	1.000
Tuberculosis, %	0.5	1.4	0.321	2.2	1.7	0.794	0.7	7.0	< 0.001
Pneumonia, %	18.9	38.6	< 0.001	11.8	27.8	< 0.001	5.5	12.2	0.009
Allergic rhinitis, %	3.6	15.7	< 0.001	5.6	21.9	< 0.001	6.9	20.9	< 0.001

^a^ Respondents with BA are respondents who reported wheezing symptoms at the ATS Respiratory Symptoms Questionnaire

Percentages are calculated from the number of respondents with valid data (valid N). *P*-values are for the 2-sided comparison (Chi-square tests) of rates between respondents with and without BA

## Discussion

The CORE study was conducted to evaluate the prevalence and burden of BA in three CIS countries using a standardized methodology. Our study showed that the prevalence of previous BA diagnosis (doctor diagnosed asthma) was 12.5, 19.0 and 26.8 per 1000 in Ukraine, Kazakhstan, and Azerbaijan, respectively, but the prevalence of BA symptoms (wheezing) identified during the study was much higher, 74.4, 254.8 and 123.4 per 1000, respectively. There were between country variations in the prevalence of wheezing; possible reasons for them may be a topic for future research. The number of respondents having wheezing/whistling during more than 5 years, based on ATS Respiratory Symptoms Questionnaire, was approximately 2–3.5-fold higher than the number of respondents who reported doctor diagnosed asthma. These findings underline that people with wheezing may have underdiagnosed BA and the majority of respondents could have been diagnosed with asthma for the first time during the study.

Statistically significant relationship was shown between smoking, overweight/obesity, dusty work and allergic rhinitis and BA symptoms (wheezing) in three countries. Co-morbidities were significantly more frequent in respondents with BA compared to those without BA.

In all studied countries the prevalence of doctor diagnosed asthma and wheezing had a tendency to increase with age (peak prevalence occurred at the age group 65 years and older in Ukraine and Kazakhstan, and 40–65 years old in Azerbaijan). However, this finding can be related to greater opportunity to be diagnosed with asthma in older people and to the fact that the study collected data on wheezing ever experienced by a respondents in their life, not current or recent wheezing. There is no clear relationship between aging and prevalence of BA in adult population in other studies [20].

In all three countries doctor diagnosed asthma and wheezing were slightly more prevalent in men than in women. Some studies also confirm this observation [19], but differences were not significant.

As for ethnic-related differences in prevalence of BA (Kazakhstan), a trend to higher prevalence of doctor diagnosed asthma and lower prevalence of wheezing symptoms in Asians compared to Caucasians was observed. In some other studies, Asians had a greater risk of asthma than Caucasians [24]. However, the number of respondents included in the prevalence calculation is quite small, the differences in this study are minor and it seems to be impossible to estimate whether they are due to national features or random observation.

### Comparison with published literature

The prevalence of doctor diagnosed asthma in this study (12.5, 19.0 and 26.8 per 1000 in Ukraine, Kazakhstan, and Azerbaijan, respectively) was significantly lower than the global estimation reported by To et al. [5] (43 per 1000); however, the values reported by To et al. for post-soviet countries (28 per 1000 in Ukraine; 14 per 1000 in Kazakhstan, and 25 per 1000 in Russia) [5] are closer to our results than the global prevalence rates. The prevalence of doctor diagnosed asthma in this study was lower than in the Russian GARD study [6] (69 per 1000), but the differences in methodology across studies may lead to the variability in the results. Relatively lower rate of BA diagnosis in the studied countries could be related to various factors. In the CIS countries lack of incentive for physicians to diagnose asthma may exist, because asthma medications are reimbursed for patients; at the same time, the funding of asthma management from the government is limited, that may force health authorities to regulate the number of asthma patients registered in primary and specialty care. The other factor which might play a role is the limited knowledge about asthma and its awareness between healthcare workers. The third reason of asthma underdiagnosis may be related to a public stigma (asthma is often believed to be a bad disease, people are afraid of inhalers and refuse to use them). The respondents in our study could have lack of knowledge as to “bronchial asthma” as the same as “asthma” and what an “exacerbation” exactly means; these factors could also affect the observed results.

Prevalence estimates of wheezing symptoms in our study was 74.4 per 1000 in Ukraine, 254.8 per 1000 in Kazakhstan and 123.4 per 1000 in Azerbaijan. The value in Kazakhstan was close to that obtained in the Russian GARD study [6] (257 per 1000). In most of the published studies wheezing was evaluated during last 12 months (not ever in life), including To et al. (86 per 1000) [5]; therefore, the results cannot be compared. Another important issue is that all wheezing is not asthma, so, the real difference between the prevalence of BA diagnosed by a doctor and BA revealed in this study cannot be stablished.

### Co-morbidities and risk factors

The role of co-morbidities in asthmatic patients is an important question. A large meta-analysis [25] previously showed that BA is associated with significantly higher co-morbidities including cardio−/cerebrovascular diseases, obesity, hypertension, diabetes, psychiatric and neurological co-morbidities, gut and urinary conditions, cancer, and respiratory problems other than asthma. Respiratory co-morbidities are found to be five times more prevalent in asthma than in non-asthma patients. Also it has been demonstrated that asthmatic subjects with co-morbid hypertension display evidence of greater asthma morbidity [26]. In our study the rate of hypertension, cardiovascular diseases, abnormal blood lipids and a history of pneumonia was significantly higher among respondents with BA compared to those without BA that is in line with published literature. However, taking into account that persons who had contraindication(s) to spirometry were not enrolled to the study, the overall rate of some diseases could be underestimated in this study (e.g. severe hypertension, myocardial infarction, other cardiovascular conditions, other severe diseases).

One of the most frequent co-morbidity in asthma patients is allergic rhinitis. Strong relationship between allergic rhinitis and BA has been established. In previously published epidemiological studies, 30 to 80% of asthmatic patients reported allergic rhinitis, and the real value can be higher [27, 28]. In our study allergic rhinitis was observed in 16–22% respondents with BA that is lower than overall rate reported in literature, but nevertheless exceeds the value observed in the respondents without BA. Additionally, allergic rhinitis was one of the risk factors for BA that is in line with published data [29].

The association of BA with various risk factors have been widely studied. As expected, smoking, overweight/obesity, dusty work, allergic rhinitis were the risk factors with significant association with BA symptoms in all three study courtiers (and tuberculosis in anamnesis in Azerbaijan). It is well known that modifiable factors such as obesity and smoking have been associated with the development of asthma [14, 30–41]. At the same time, some studies demonstrated that dusty work can be associated with an elevated risk of asthma [42, 43], as well as in a farming environment [44].

### Strengths and limitations

This study has several strengths. It is a multi-national, cross sectional, population-based study with a sample size using consistent methodology across all countries, providing a standardised measure of prevalence in the CIS countries. In addition, the case definition of BA used is based on both (i) doctor diagnosed asthma and (ii) wheezing symptoms using a validated questionnaire. This study could facilitate the recognition of BA in CIS countries using a standardized methodological approach and will allow preparation of interventions to optimize management of patients with BA.

We acknowledge that the current study has several limitations. The method of districts and streets sampling may not ensure completely random selection of streets and participants. Relatively small number of BA patients limits the analysis of risk factors associated with BA and can limit the power for specific type of within-the-city analysis, such as detailed subgroup analyses. The subjectivity of the diagnostic criteria based on symptoms can lead to over or underdiagnosis. Spirometry was performed in the study (for COPD diagnosis) but its results were not used for confirmation or exclusion of BA diagnosis. Respondents with contraindications for spirometry (usually severe pathologies) were not enrolled in the study that may lead to underestimating of BA prevalence.

The city population may not be representative of each country in general, because risk factors and healthcare provision (including the availability of medical care) may vary within the country. Particularly, results may only reflect the urban agglomerations studies and not represent the whole country, because rural areas could have different medical care level or living and working conditions for people.

Some data can be incomplete due to lack of relevant information from participants, as a lot of study data were collected from respondents themselves. The number of respondents included in the prevalence calculation, especially for age- and ethnicity subgroups, is quite small, that could be another limitation of the study.

## Conclusions

In conclusion, in Ukraine, Kazakhstan and Azerbaijan the prevalence of doctor diagnosed asthma is significantly lower than prevalence of wheezing symptoms underlining that BA is likely to be underreported in these countries. The information provided in this paper will be helpful for healthcare policy makers in CIS countries to instruct BA management strategies and allocate healthcare resources accordingly.

## Abbreviations

ATS: American Thoracic Society
BA: Bronchial Asthma
BMI: Body mass index
CI: Confidence interval
CIS: Commonwealth of Independent States
COPD: Chronic Obstructive Pulmonary Disease
GARD: Global Alliance against Chronic Respiratory Diseases
GINA: Global Initiative for Asthma
GSK: GlaxoSmithKline
OR: Odds ratio
SD: Standard deviation
